# Editorial: Ras and Other GTPases in Cancer: From Basic to Applied Research

**DOI:** 10.3389/fmolb.2021.804818

**Published:** 2021-11-29

**Authors:** Kwang-jin Cho, Jin Rui Liang, Piero Crespo, Veronica Aran

**Affiliations:** ^1^ Department of Biochemistry and Molecular Biology, Boonshoft School of Medicine, Wright State University, Dayton, OH, United States; ^2^ Department of Biology, ETH Zürich, Zürich, Switzerland; ^3^ Instituto de Biomedicina y Biotecnología de Cantabria (IBBTEC), Consejo Superior de Investigaciones Científicas (CSIC)-Universidad de Cantabria, Santander, Spain; ^4^ Instituto Estadual do Cérebro Paulo Niemeyer, Rio de Janeiro, Brazil

**Keywords:** small GTPases, Ras, Rho, Rap, RAS mutations, SmgGDS, cmp4, cancer therapy

In the current Research Topic (RT), we provide insights on the small GTPase biology, from protein synthesis to disease, by presenting a Research Topic of original and review articles describing distinct GTPases, with special attention to RAS proteins. The RAS superfamily comprises a large family of small GTPases, where the members of the RAS, RHO, and RAB families are the most well-characterized. These small proteins cycle between a GDP-bound inactive and a GTP-bound active state, thus mediating several cellular processes such as intracellular trafficking, cytoskeletal organization, cell migration, proliferation, differentiation, and gene expression (Bos 2018; Gray et al., 2020).

RAS is considered a central protooncogene in cancer. There are different RAS proteins (K-RAS, N-RAS, and H-RAS) and gain-of-function missense mutations in RAS genes are frequently found in a variety of tumors (Hobbs et al., 2016). Although there are different RAS isoforms, most research to date have concentrated their efforts in studying K-RAS, since it is the most mutated isoform in cancer. In addition, the K-RAS gene undergoes alternative splicing generating the splicing variants K-RAS4A and K-RAS4B. In this RT, Aran discusses the roles of both splicing variants in cancer and since K-RAS4A, the under-studied splicing variant, shares the same oncogenic point mutations with K-RAS4B and has strong transforming capability, the author emphasizes the importance of investigating K-RAS4A in RAS-driven cancer and developing anti-RAS therapies.

RAS undergoes several posttranslational modifications (PTMs) that facilitate its attachment to membranes, where it drives its signal transduction. The cysteine in the C-terminal CAAX motif (where A is aliphatic and X is any amino acid) is first prenylated, allowing the cytosolic RAS proteins to bind the ER, where the -AAX residues are cleaved and the now C-terminal prenylated cysteine is methylated. H-, N- and K-RAS4A are also palmitoylated. Busquets-Hernandez and Triola contribute a review on the role of lipid modifications, specifically palmitoylation, on the regulation of RAS activity; how palmitoylation orchestrates RAS distribution over different subcellular compartments and its compartmentalization within membrane subdomains, and how it impacts on RAS functions. The authors also discuss the potential of these to be translated into therapeutics.

Brandt et al. provide a review on the role of Small GTP-binding protein GDP dissociation stimulator (SmgGDS) as major regulators of the prenylation, post-prenylation processing, and trafficking of RAS and RHO small GTPase family members. The authors further provide new strategies for therapeutic targeting of SmgGDS in cancer, involving splice-switching oligonucleotides and peptide inhibitors. Moreover, the signal transduction and subcellular localization of RAS proteins can be further regulated by reversible PTMs at their G4 and G5 motifs, including S-oxygenation, S-nitrosylation, monoubiquitylation, acetylation, and methylation. Osaka et al. provide a review discussing the mechanisms of these PTMs and propose that targeting these PTM mechanisms can be a good starting point for developing a new therapeutic approach for RAS-driven cancers.

Recently, direct K-RASG12C mutant inhibitors showed promising outcomes in clinical trials (Canon et al., 2019; Hallin et al., 2020), but since this mutant is found only in a small portion of K-RAS-driven cancers, pan-K-RAS therapies are still needed. Since most functional RAS proteins localize to the plasma membrane (PM), targeting the RAS-PM interaction represents a potential alternative strategy to disrupt RAS signaling activity. Zhou et al., in their review, summarizes the latest mechanistic insights on how different RAS isoforms undergo spatial segregation with different PM lipid species and how this could impact on the recruitment of their respective effectors and activation of different downstream signaling pathways. The authors further discuss the possibility of targeting RAS nanoclusters as a potential therapeutic approach to treat RAS pathologies. Moreover, Henkels et al. described how pharmacological agents disrupting K-RAS-PM interaction could be beneficial to block oncogenic K-RAS activity, thus representing clinical utility. K-RAS membrane organization is dependent on Calmodulin (CaM) and has significant impact on cancer stem cell tumorigenesis. Here, Okutachi et al. describes a novel CaM inhibitor, Calmirasone1, which has higher on-target inhibition on K-RAS compared to its off-target substrates including H-RAS and B-RAF. This discovery has exciting future applications for the interrogation of the cancer biology of CaM-associated K-RAS activities.

Tisi et al. describe a novel RAS inhibitor, cmp4, which exerts antiproliferative effects on cancer cells resistant to EGFR-aimed therapy. Cmp4 binds an extended Switch II pocket on H-RAS and K-RAS and induces a conformational change that abrogates guanine nucleotide exchange and impedes RAS effector binding. In this respect, cmp4 could provide a template for future drugs exploiting this promising mechanism of action.

Bartolacci et al. reviewed the recent advances concerning the relationship between RAS and lipid metabolism in cancer, describing how lipids and oxidative stress can either promote or sensitize to ferroptosis (i.e., an iron-dependent programmed cell death defined by the existence of oxidative stress and lipid peroxidation) RAS-driven cancers, which is still a controversial subject. The authors argue that RAS mutations have tissue-specific effects on metabolism, probably due to the intrinsic metabolic wiring present in distinct tumor types, and that the combination between ferroptosis inducers with existing chemotherapeutic agents, could be of potential clinical benefit.

Finally, RAS related proteins (RAP) are members of the RAS superfamily, sharing 50–60% sequence homology with RAS, and being involved in cell adhesion, migration, and polarity (Bokoch 1993). There are five different RAP family members, which are shown to be involved in the tumorigenesis of multiple cancer types (Bokoch 1993; Simanshu et al., 2017). Kumari et al. utilize authoritative multi-omics databases to investigate the association of RAP gene family expression with molecular and clinicopathological features in hepatocellular carcinoma (HCC). Their study reveals significant associations of one of the RAP family members, RAP2A expression with several HCC pathways, including cell cycle-related pathways and metabolic pathways, suggesting RAP2A as a therapeutic target and prognostic biomarker in HCC.

Overall, this RT discusses the role of small GTPases in carcinogenesis and up-to-date strategies to block their oncogenic activities in cancer. We hope that the selected articles will inspire and motivate basic and clinical research scientists to further investigate several unanswered questions concerning the GTPases world. Despite being small proteins in size, their biological importance is substantial.

## References

[B1] Bokoch G. M. (1993). Biology of the Rap Proteins, Members of the Ras Superfamily of GTP-Binding Proteins. Biochem. J. 289 ( Pt 1) (Pt 1), 17–24. 10.1042/bj2890017 8424755 PMC1132124

[B2] Bos J. L. (2018). From Ras to Rap and Back, a Journey of 35 Years. Cold Spring Harb Perspect. Med. 8. 10.1101/cshperspect.a031468 PMC5793741 28778969

[B3] Canon J. Rex K. Saiki A. Y. Mohr C. Cooke K. Bagal D. (2019). The Clinical KRAS(G12C) Inhibitor AMG 510 Drives Anti-tumour Immunity. Nature 575, 217–223. 10.1038/s41586-019-1694-1 31666701

[B4] Gray J. L. Delft F. Brennan P. E. (2020). Targeting the Small GTPase Superfamily through Their Regulatory Proteins. Angew. Chem. Int. Ed. 59, 6342–6366. 10.1002/anie.201900585 PMC7204875 30869179

[B5] Hallin J. Engstrom L. D. Hargis L. Calinisan A. Aranda R. Briere D. M. (2020). The KRASG12C Inhibitor MRTX849 Provides Insight toward Therapeutic Susceptibility of KRAS-Mutant Cancers in Mouse Models and Patients. Cancer Discov. 10, 54–71. 10.1158/2159-8290.cd-19-1167 31658955 PMC6954325

[B6] Hobbs G. A. Der C. J. Rossman K. L. (2016). RAS Isoforms and Mutations in Cancer at a Glance. J. Cel Sci 129, 1287–1292. 10.1242/jcs.182873 PMC4869631 26985062

[B7] Simanshu D. K. Nissley D. V. McCormick F. (2017). RAS Proteins and Their Regulators in Human Disease. Cell 170, 17–33. 10.1016/j.cell.2017.06.009 28666118 PMC5555610

